# The recent introduction of *Angiostrongylus cantonensis* and its intermediate host *Achatina fulica* into Guadeloupe detected by phylogenetic analyses

**DOI:** 10.1186/s13071-023-05872-4

**Published:** 2023-08-10

**Authors:** Gelixa Gamiette, Séverine Ferdinand, David Couvin, Céline Dard, Antoine Talarmin

**Affiliations:** 1 Réservoir et Diversité des Pathogènes, Unité Transmission, Institut Pasteur de Guadeloupe, Pointe-à-Pitre, France; 2 Institute for Advanced Biosciences, Team Host-Pathogen Interactions and Immunity to Infection, INSERM U1209—CNRS UMR5309, Université Grenoble Alpes, Grenoble, France

**Keywords:** *Angiostrongylus cantonensis*, *Achatina fulica*, Guadeloupe, Phylogeny, Cytochrome C, Cytochrome B, 16S ribosomal RNA

## Abstract

**Background:**

*Angiostrongylus cantonensis* (rat lungworm) is the main pathogen responsible for eosinophilic meningitis in humans. One of its intermediate snail hosts, *Achatina fulica*, was already present in many countries around the world before it appeared in the West Indies in the late 1980s. In the French territories in the Caribbean and northern South America, the first cases of human neuroangiostrongyliasis were reported in Martinique, Guadeloupe and French Guiana in 2002, 2013 and 2017, respectively. In order to better characterize angiostrongyliasis in Guadeloupe, particularly its geographical origin and route of introduction, we undertook molecular characterization of adult worms of *Angiostrongylus cantonensis* and its intermediate host *Achatina fulica*.

**Methods:**

Genomic DNA of adult *Angiostrongylus cantonensis* and *Achatina fulica* was extracted and amplified by polymerase chain reaction (PCR) targeting the mitochondrial genes cytochrome B and C for *A. cantonensis* and 16S ribosomal RNA for *A. fulica*. The PCR products were sequenced and studied by phylogenetic analysis.

**Results:**

Cytochrome B and cytochrome C molecular markers indicate a monophyletic lineage of *A. cantonensis* adult worms in Guadeloupe. Two sequences of *A. fulica* were identified.

**Conclusions:**

These results confirm the recent introduction of both *Angiostrongylus cantonensis* and *Achatina fulica* into Guadeloupe. *Achatina fulica* in Guadeloupe shares a common origin with those in Barbados and New Caledonia, while *Angiostrongylus cantonensis* in Guadeloupe shares a common origin with those in Brazil, Hawaii and Japan.

**Graphical Abstract:**

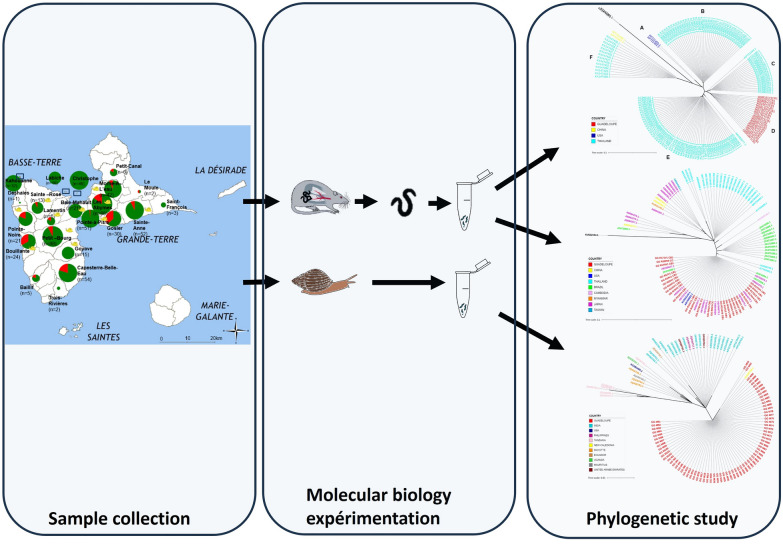

## Background

*Angiostrongylus* spp. are parasitic worms in the superfamily Metastrongyloidea and the family Angiostrongylidae. There are 22 species in the genus *Angiostrongylus* [1, 2], but only *Angiostrongylus cantonensis* and *Angiostrongylus costaricensis* are known to infect humans. In this work, we focused on *A. cantonensis*.

*A. cantonensis* was first described in 1955. Its life cycle is complex and involves rats as definitive hosts and molluscs as intermediate hosts [3]. The adult worms live in the pulmonary arteries and in the right heart of the rat, where the females lay their eggs. The eggs travel passively in the blood to the lungs, where they hatch. Then stage-1 larvae (L1) emerge, travel up the trachea, are swallowed and travel down the digestive tract before being released in the feces. The larvae are then ingested by a gastropod mollusk that feeds on the feces. In the mollusk, the larvae undergo two successive molts and reach the L3 stage, when they become infective. The definitive host is infected by ingesting intermediate hosts carrying L3 larvae. Animals that feed on gastropod mollusks may also play a role in the passive transport of infective L3 larvae, as paratenic hosts of *A. cantonensis* in which it cannot complete its life cycle. These paratenic hosts include terrestrial planaria, centipedes, fresh and brackish water crustaceans (shrimp, mangrove crabs), land crabs (including coconut crabs), amphibians (toads, frogs), and reptiles (monitor lizards) [4]. Humans are accidental hosts and also do not allow completion of the parasite’s life cycle. Infection occurs by consumption of raw or undercooked intermediate or paratenic hosts.

First described in China [5], *A. cantonensis* is now present in many countries, with a wide distribution in tropical regions. It was reported in the Indian Ocean in the 1960s [6, 7], and has been reported in the Pacific region [8] and Africa since the 1970s [9], and in the Americas and the Caribbean since the 1980s [10–12]. The spread of the parasite throughout the world is linked to the spread of its final or intermediate hosts; the giant African snail *Achatina fulica* may have played a role in its dispersal [13], although rats may have been more important in this [14]. These invasive species have dispersed into new environments, probably as a result of increased international trade as well as climate change [15].

The main signs and symptoms of infection with *A. cantonensis* L3 larvae are severe headache and paresthesia, reflecting the inflammation due to the presence of the larvae, which exhibit central nervous system tropism. Neurologic manifestations include eosinophilic meningitis, encephalitis/encephalomyelitis, radiculitis, cranial nerve abnormalities, and ataxia [16]. Most infections are mild and resolve without treatment, but some are fatal, especially in young children, who are at the highest risk [17]. *Angiostrongylus cantonensis* is now the main infectious cause of eosinophilic meningitis worldwide, although neuroangiostrongyliasis remains a relatively rare disease, with approximately 3000 cases documented in the international literature [18, 19]. Human cases of neuroangiostrongyliasis have been reported mainly from Southeast Asia (China and Thailand) [19, 20], islands in the Pacific [21] and in the Indian Ocean [22], the Greater Antilles (Cuba, Jamaica) [12], and, more recently, in Brazil [23].

The first human cases of neuroangiostrongyliasis in the French overseas territories in the Americas were diagnosed in Martinique in 2002, Guadeloupe in 2013, and the Guiana Shield in 2017 [24]. Since those reports, numerous cases have been reported in Martinique and Guadeloupe [25]. The emergence of *Angiostrongylus cantonensis* in the French West Indies may be due to the recent introduction of *Achatina fulica* into this region, as *A. fulica* is a common intermediate host involved in *A. cantonensis-*related infections. The range of this gastropod, which is native to East Africa and is a pest of agricultural crops, has expanded, and its presence has been reported in Asia, South America and Florida in North America. It was first reported in the Caribbean, and particularly in the French West Indies, at the end of the 1980s [26]; however, little information is available about the intermediate hosts of *A. cantonensis* in the French West Indies.

After the emergence of cases of neuroangiostrongyliasis in Guadeloupe, *Achatina fulica* were collected in 7 municipalities on Guadeloupe (Vieux-Habitant, Basse-Terre, Trois-Rivières, Saint-Claude, Lamentin, Saint-Anne and Capesterre de Marie-Galante) to evaluate the prevalence of *Angiostrongylus cantonensis*. Approximately 38% of these snails were infected with *A. cantonensis* (unpublished data). Although their presence has been reported for several years in the Caribbean, few molecular data are available for either *Angiostrongylus cantonensis* or *Achatina fulica* in the region.

To determine the geographic origin and route of introduction of *Angiostrongylus cantonensis*, and to better define the role of *Achatina fulica* in the emergence of *A. cantonensis* in Guadeloupe, we undertook molecular characterization of both *A. cantonensis* and *A. fulica*.

## Methods

### Sampling

**Fig. 1:**
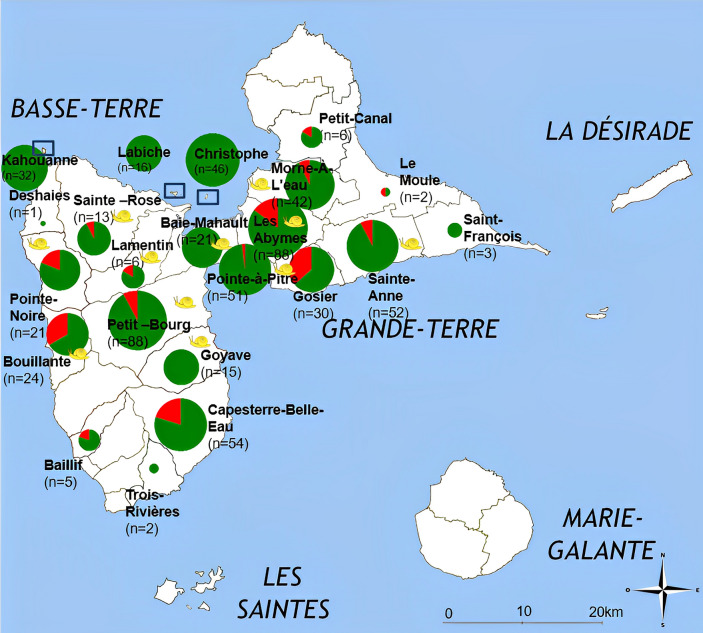
Geographic distribution of *Angiostrongylus cantonensis* in rats on Guadeloupe and outlying islets. Red sectors on the pie charts indicate positive cases and green sectors negative cases. The yellow snails indicate the sites at which *Achatina fulica* were captured*.* The original map is available under Creative Commons license https://commons.wikimedia.org/wiki/File:Guadeloupe_map.JPG

Between 2013 and 2021, a total of 618 wild rats (*Rattus rattus* and *Rattus norvegicus*) were captured in 19 municipalities on the main islands (Basse-Terre and Grande-Terre) and on three islets of Guadeloupe (Fig. 1). The lungs of 349 of the rats captured by the French Agricultural Research Centre for International Development in 2013, of 94 rats captured by Guadeloupe National Park personnel in 2020, and of 175 rats that we captured in 2021, were dissected. Adult *Angiostrongylus* worms extracted from the rat lungs were individually preserved in absolute ethanol and stored at − 20 °C until use. In 2022, a total of 105 *A. fulica* were collected from 10 sites on Guadeloupe and frozen at − 20 °C until DNA extraction.

### DNA extraction

Total DNA from adult *A. cantonensis* worms was extracted individually with the cetyltrimethylammonium bromide technique described previously [27], with slight modifications. The incubation temperature was increased to 65 °C for 1 h; the addition of isopropanol was adjusted to 0.6 µL per microlitre of supernatant; the centrifugation time after the addition of isopropanol was increased to 30 min; and the samples were dried at room temperature for 40 min before suspending the DNA in 40 µL of RNase-free water. *Achatina fulica* DNA was extracted from a piece of the foot measuring approximately 8 mm^3^, in accordance with the DNA extraction conditions described previously [28].

### Molecular characterization of *Angiostrongylus cantonensis* and *Achatina fulica*

The identity of the worms was confirmed by amplification of the internal transcribed spacer 1 (ITS1), with *A. cantonensis*-specific primers (ITS1-F1 and ITS1-R1) used for real-time PCR [29].

The genetic diversity of *A. cantonensis* was characterized after PCR amplification of the mitochondrial cytochrome B (cytB) and cytochrome C (cytC) genes by conventional PCR. PCR was performed with the primers cytb-F, 5′-TGAATAGACAGAATTTTAAGAG-3′, and cytb-R, 5′-ATCAACTTAACATTACAGAAAC-3*'*, described previously [30] for cytB, and the newly designed primers 5′-TTAGTTTRCATTGTGCTGG-3' and 5′-CATCAAAGACTAATACCAG-3*'* for cytC. The expected amplicon sizes were 853 base pairs (bp) for cytB and 748 bp for cytC. PCR was performed in a final volume of 50 μL under the following conditions: 5 μL of 10X buffer; 2 μL of 25 mM MgCl_2_; 2 μL of 5 mM deoxynucleotide triphosphate; 2 μL of bovine serum albumin; 3 μL of dimethyl sulphoxide; 0.25 μL of Taq DNA polymerase at 5U/μL (Eurobiotaq, Courtaboeuf, France); 1 μL of each of the two primers at 20 μM; 28.75 μL of ultrapure water; and 5 μL of DNA.

The genetic diversity of *A. fulica* was characterized by amplification of the 16S ribosomal RNA (rRNA)_gene by standard PCR. The primers and PCR conditions described previously generated a fragment of 293 bp [28]. The PCR reaction and the size of the resulting fragments were monitored by migration on 1.5% agarose gel in 1× Tris–acetate-ethylenediaminetetraacetic acid buffer. A total of 10 μL of PCR products mixed with 2 μL of 5× bromophenol blue was placed in each well. Migration was performed in 1× Tris–acetate-ethylenediaminetetraacetic acid buffer at 90 V for 45 min.

### Sequencing and phylogeny reconstruction

Amplicons were purified and sequenced by the Sanger method. The experimental sequences obtained from the mitochondrial cytB and cytC genes of *Angiostrongylus cantonensis* and the 16S rRNA gene of *Achatina fulica* were first examined for quality control and compared with published sequences in the National Center for Biotechnology Information (NCBI) GenBank database. The 71 new experimental sequences generated for this study are available in GenBank.

Sequences were trimmed with BioEdit [31], and the subsequent steps of the analysis (presented below) were processed on the Galaxy KaruBioNet interface [32]. Phylogenetic reconstruction based on the maximum likelihood method (1000 bootstrap) was performed with RAxML v8 [33] from the multiple sequence alignments obtained with MAFFT software version 7 [34]. The maximum likelihood phylogenetic trees were then drawn with iTOL [35].

### Statistical analysis

A chi-squared test was used to compare the prevalence of *A. cantonensis* infection in the two rat species (*R. rattus* and *R. norvegicus*), and Student’s* t*-test was used to compare the prevalence of *A. cantonensis* among municipalities on Guadeloupe. Odds ratios and 95% confidence intervals were calculated. R software version 4.1.2 (2021-11-01) was used for statistical analysis, and *P-*values < 0.05 were considered significant.

## Results

### Survey of *A. cantonensis* adult worms in definitive hosts in Guadeloupe

Of the 32 municipalities on Guadeloupe, 19 were investigated. We found that 15.5% (28/181) of *R. norvegicus* and 11.2% of *R. rattus* (38/338) had adult worms in their pulmonary arteries, representing 12.7% of all of the rats examined. The difference between the two species was not statistically significant (*χ*^2^ = 1.8975, *df* = 1, *P* = 0.1684). The frequency of rats carrying *A. cantonensis* varied from one municipality to another (Fig. 1). The parasite was found in both Basse-Terre (seven of eight municipalities) and Grande Terre (seven of 11 municipalities); however, it was present in higher proportions in Bouillante, Gosier and Moule. It was not carried by rats caught in Trois-Rivières, Goyave, Baie-Mahault or Saint François (Fig. 1). Statistically significant differences were observed between the numbers of rats that were positive and those that were negative for *A. cantonensis* in the municipalities (*t* = − 3.9645, *df* = 13, *P* = 0.001617). None of the rats caught on the three offshore islets (Kahouanne, Labiche and Christophe) were infected with *A. cantonensis.*

### Phylogenetic analysis of *A. cantonensis* adult worms based on cytB sequences

**Fig. 2:**
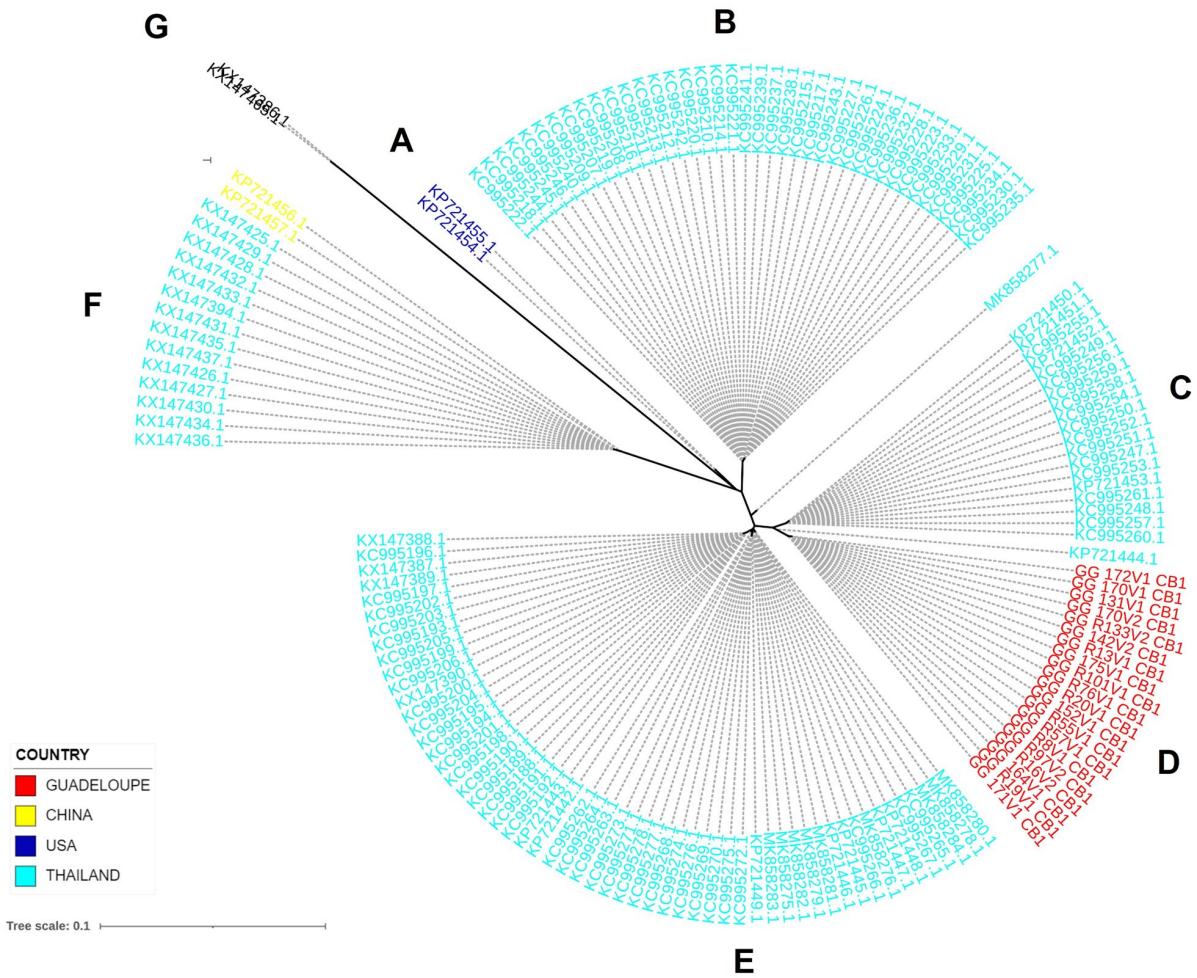
Maximum likelihood phylogenetic unrooted tree based on cytochrome B (cytB) sequences of *Angiostrongylus cantonensis* adult worms. Two sequences of *Angiostrongylus malaysiensis* were used as an outgroup. Letters indicate identified clusters. Labels are coloured according to the country of isolation. The scale indicates the length of the branches representing the number of nucleotide substitutions per site

The phylogenetic tree (Fig. 2), based on partial sequences of cytB of *A. cantonensis* (668 bp), was constructed with the maximum likelihood method. Comparison of the 20 sequences from Guadeloupe with 137 sequences previously deposited in the NCBI GenBank database revealed the presence of six clusters, recorded as A, B, C, D, E and F (Fig. 2). Branch G was an outgroup composed of two sequences of *Angiostrongylus malaysiensis.* All the individuals from Guadeloupe were located on branch D, indicating a single monomorphic cytB pattern, specific to Guadeloupe. All the profiles described in Guadeloupe were distinct from those previously described in China, Thailand, and the USA, but the cluster from Guadeloupe (D) was close to cluster C. Individuals from Thailand were overrepresented, as they represented 85% of the individuals on this tree. They constituted four clusters (B, C, E, F), three of which are composed exclusively of individuals from Thailand (B, C, E). One (cluster F), the furthest from the Guadeloupe cluster, is shared by Chinese and Thai individuals. Cluster A is found only in Hawaii.

### Phylogenetic analysis of *A. cantonensis* adult worms based on cytC sequences

**Fig. 3:**
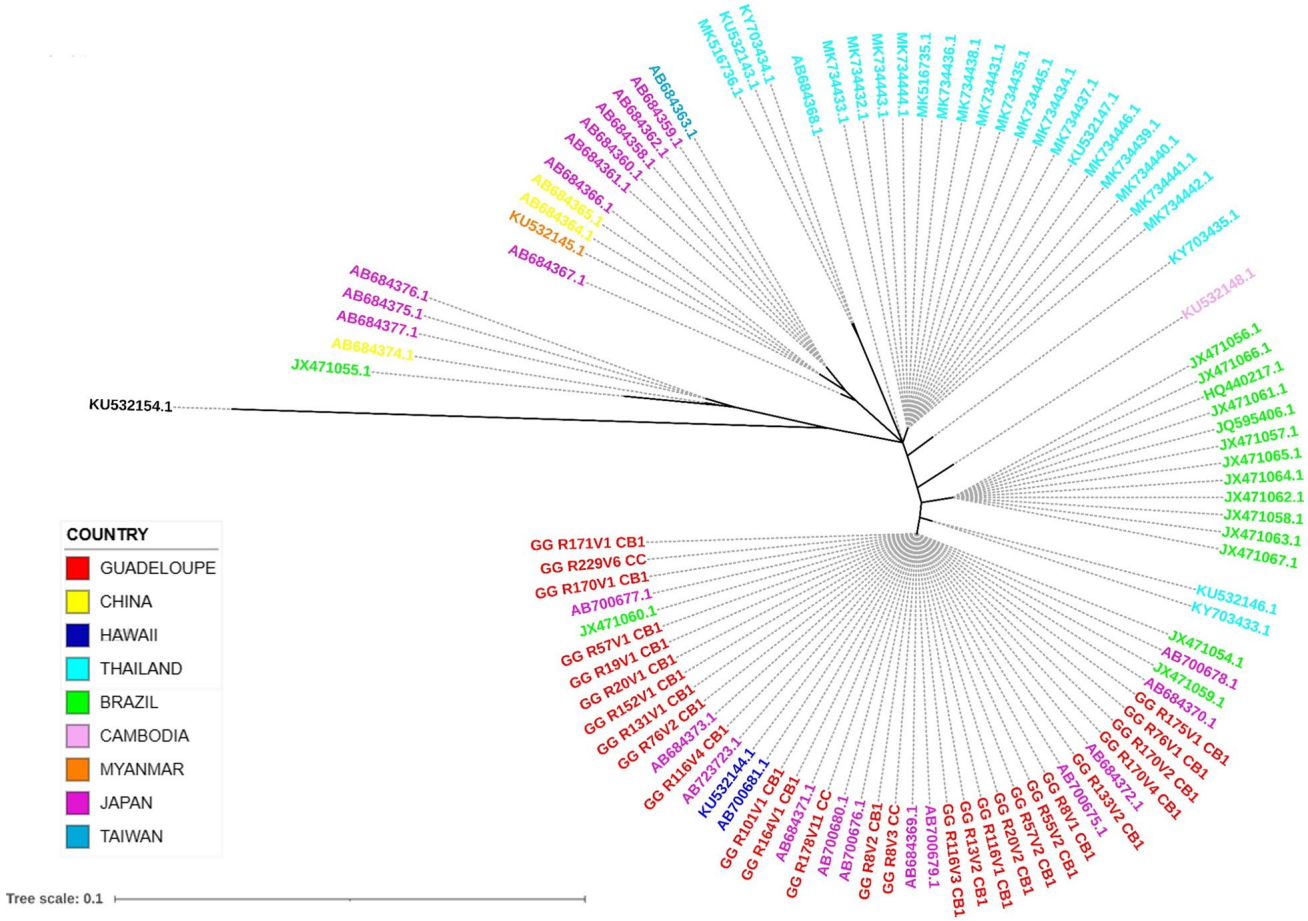
Maximum-likelihood phylogenetic unrooted tree based on cytochrome C (cytC) sequences of adult *Angiostrongylus cantonensis* worms. *Angiostrongylus malaysiensis* was used as an outgroup. Labels are coloured according to the area of isolation. The scale indicates the length of the branches given as the number of nucleotide substitutions per site

The phylogenetic tree (Fig. 3) based on the partial sequence of cytC of *A. cantonensis* (351 pb) was constructed with the same technique as above. A total of 27 cytC sequences of individual adult *A. cantonensis* from Guadeloupe were compared with 72 cytC gene sequences in GenBank. One sequence of *A. malaysiensis* (KU532154) was included as an outgroup. Phylogenetic reconstruction showed the presence of 13 branches. Individual adult worms from Guadeloupe appeared to be monomorphic for this marker and their sequences were identical to 13 Japanese, two Hawaiian, and three Brazilian sequences. All of these individuals clustered on the same branch. At least two sequences were found for worms from all three areas, whereas only one sequence was seen in Guadeloupe.

### Phylogeny of *A. fulica* by the 16S rRNA partial gene sequences

**Fig. 4:**
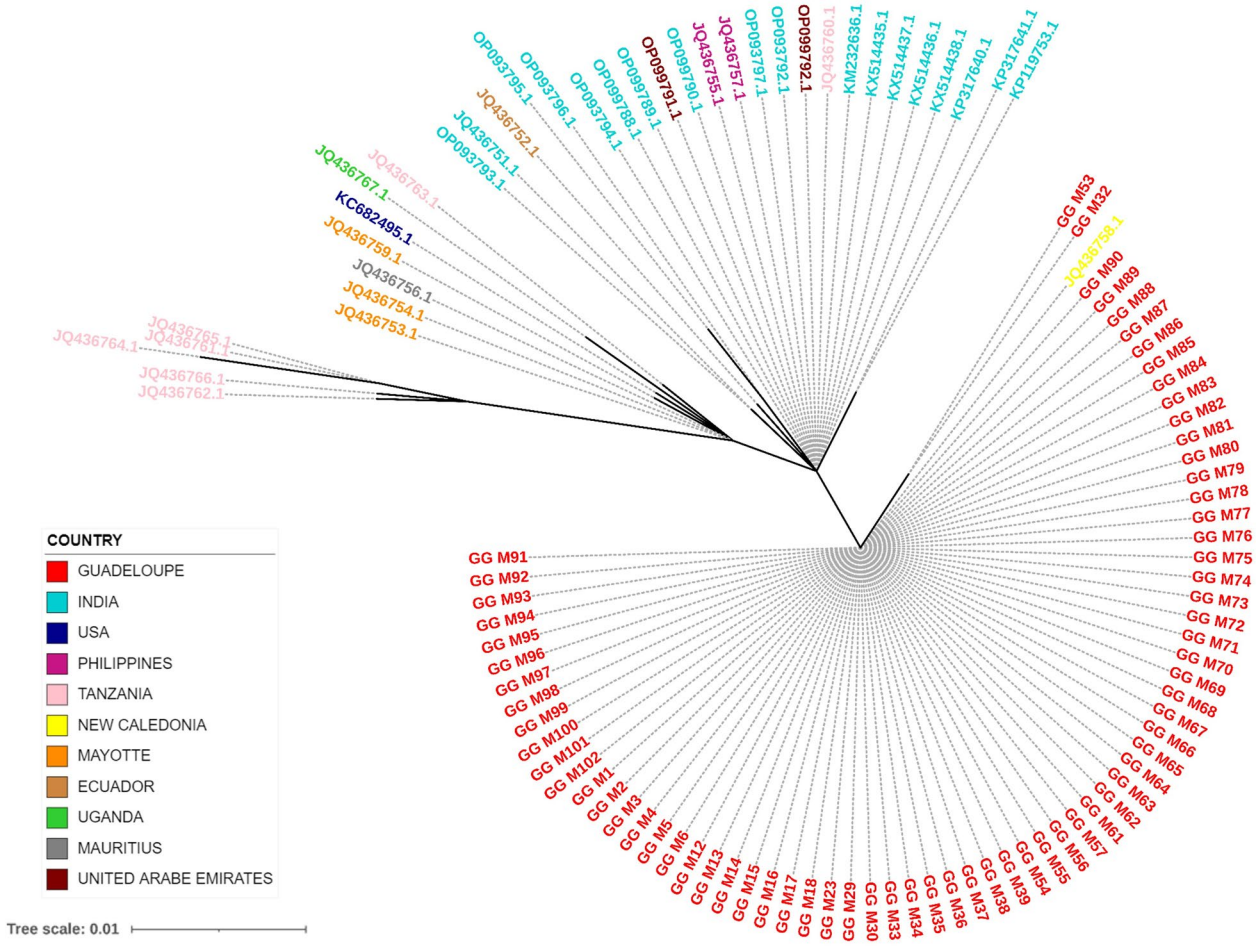
Maximum-likelihood phylogenetic unrooted tree based on the rRNA 16S gene of *Achatina fulica* snails. Labels are coloured according to the country or area of isolation. The scale indicates the length of the branches given by the amount of genetic change in nucleotide substitutions per site

The phylogenetic tree (Fig. 4) based on the partial sequence of the 16S rRNA gene of *A. fulica* (243 pb) was constructed with the same technique as above. A total of seventy-one 16S rRNA gene sequences of *A. fulica* from Guadeloupe were compared with the 37 sequences from 10 countries and areas in the NCBI GenBank database.

Among the 71 sequences from 10 localities in Guadeloupe, two different yet closely related sequences were found, which differed in the position of one nucleotide in our alignment. The largest group consisted of 69 individuals, which shared a sequence with one individual from New Caledonia (JQ436758.1). In the other group, two individuals shared a sequence that has not been described previously (Fig. 4). These sequences (GG_M32: OP962241 and GG_M53: OP962249) have a uracil while the others have a thymine at position 71 of the Fontanilla et al. [28] alignment. These two individuals were collected from Lamentin and Gosier at the same time as other individuals that belonged to the larger group. The similar sequences found for Guadeloupe and New Caledonia appeared to be closer to two sequences from India (KP317641.1 and KP119753.1) than to those from Tanzania, Mayotte, Mauritius and the USA (Fig. 4). Countries and administrative areas including Tanzania, Mayotte, the United Arab Emirates and India show diversity among samples.

## Discussion

*Angiostrongylus cantonensis* is present in a number of Caribbean countries, and wild rat hosts of *A. cantonensis* have been examined in Cuba, Jamaica, Puerto Rico, Dominican Republic, Haiti, and Grenada. The prevalences of *A. cantonensis* varied from 22% in Cuba [10] to 60% in Jamaica [34], which are higher than the prevalence found in the present study for Guadeloupe (12.7%); however, the samples in those studies were smaller, ranging from 20 for Cuba to 192 for Grenada, and were probably less representative than the Guadeloupean collection. Globally, the prevalence of adult worms in wild rats ranges from 1.6%, in Thailand, to 73%, in Hawaii [36, 37]. The prevalence in our study differed between the main islands of Guadeloupe, where 12.7% (67/524) of rats were infected, and the islets, where none of the 91 rats examined was infected. This difference could have been due to the absence of *A. fulica* from the islets and its presence on Basse-Terre and Grande-Terre, where it is the species of snail most frequently encountered. The life cycle of *A. cantonensis* requires a definitive host (usually a rat) and an intermediate host (usually a snail). If one of these is absent, the parasitic cycle of *A. cantonensis* cannot be completed. *Achatina fulica* is often considered the main intermediate host of *Angiostrongylus cantonensis*, and could be one of the main species responsible for its spread worldwide [7], although this hypothesis has been questioned [14]. The emergence of human cases of neuroangiostrongyliasis on Guadeloupe some years after the first report of *Achatina fulica* there [38] suggests that the disease’s emergence was related to the introduction of these snails.

Because of the recent emergence of *A. cantonensis* infections in Guadeloupe, we expected to find few differences among the genetic sequences of individual *A. cantonensis*, especially as differences between the sequences of individuals are rare, even when they are sampled from large countries [37]. The cytB and cytC markers did not reveal polymorphism among the specimens found on Guadeloupe. This observation is consistent with the hypothesis that the parasite was recently introduced into Guadeloupe; however, the perfect identity of these two mitochondrial genes also implies that a marker for better discrimination is necessary for sequence differentiation of individuals. The results obtained here for the phylogeny of *A. cantonensis* do not clearly demonstrate the geographic origin of the parasite present in Guadeloupe. The cytC tree shows that the sequence found in Guadeloupe has also been reported in three other areas, Hawaii, Japan and Brazil, indicating that the parasite may have come from one of these, or shares a common origin with the populations found in them. As direct trade between Japan, Hawaii and Guadeloupe is rare, and the parasite has been present for a longer time in Asia than in the Americas, Brazil may have been contaminated by an individual worm from Japan or Hawaii, and a secondary contamination may have occurred in Guadeloupe as a consequence of exchanges with Brazil. However, even though there may have been only a single introduction of *A. cantonensis* into Guadeloupe, and perhaps also into Hawaii, this is probably not the case for Brazil, as the distance between the different Brazilian sequences suggests multiple arrivals of the parasite. Unfortunately, the results obtained with the cytC phylogeny could not be confirmed by the cytB phylogeny because of the geographic disparity of the published data. The tree based on cytB relates to only four countries or administrative areas: China, Hawaii, Thailand and Guadeloupe. Among these, only the cytC sequence from Hawaii was identical to that of the individual *A. cantonensis* from Guadeloupe. Unfortunately, this individual which is identical for cytC was not sequenced for cytB, so we cannot compare these worms for this latter gene. If this gene is also identical in the Hawaii and Guadeloupe individuals, a common origin would be more likely. Phylogenetic analysis based on complete mitochondrial DNA sequences would have been more discriminating and would have helped distinguish the Guadeloupe sequences from the Brazilian and Hawaiian sequences, and perhaps even the two Guadeloupean sequences. That would, however, have still been challenging, as the differences are quite small, even in a complete mitochondrial DNA sequence, especially for different areas of Southeast Asia, where the parasite is endemic [39]. The geographic origin of the Guadeloupean individuals cannot be determined because the technique has been used in very few studies and data for large parts of the world, especially Africa, are still lacking. It will therefore be difficult to affirm the geographic origin of the *A. cantonensis* individuals in Guadeloupe, even if it is unlikely that they originate from the African continent [40].

The second objective of our study was to better understand the role of *Achatina fulica* in the emergence of neuroangiostrongyliasis on Guadeloupe. Our results suggest that Guadeloupe was invaded by *A. fulica* from a single origin, belonging to the F haplotype described by Fontanilla et al. [28], which is close to that of *A. fulica* from New Caledonia and Barbados [28], although we were unable to find sequences of *A. fulica* from Barbados in GenBank. Of the 71 individuals from Guadeloupe, only two had sequences that were slightly different from those of the others. On Mayotte, a French island in the Indian Ocean which is smaller than Guadeloupe, two closely related sequences were found in three individuals. As *A. fulica* has been reported in Mayotte over a long period of time [41], even with a single introduction, genetic drift is logical. The mutation observed in the sequences of the two individuals from Guadeloupe indicates that either *A. fulica* has also been evolving since its introduction into Guadeloupe, or that the *A. fulica* with the less frequently seen sequence were also introduced in small numbers, perhaps in the same initial propagule. Previous studies reported the presence of 23 haplotypes of the 16S rRNA gene in *A. fulica* in their worldwide geographic distribution [28, 42, 43]. The C haplotype has been found in most parts of the world and is considered to be the ancestral haplotype from which the other haplotypes, notably the F haplotype, would have been derived by substitution mutation. Curiously, the *A. fulica* on Guadeloupe are different from those on Martinique, another island in the French West Indies, which is only 200 km from Guadeloupe and has numerous exchanges with Guadeloupe via air and sea. In Martinique, 100% of the snails studied belonged to haplotype C [28], which has been found in all countries and areas sampled except for Tanzania, Uganda, New Caledonia and Barbados [28]. However, sequences of *A. fulica* from Martinique were not included in Fig. 4 since none were found in Genbank. It would be interesting to study more *A. fulica* from Martinique to confirm that they all differ from those from Guadeloupe.

Because of its recent introduction into the Caribbean, *Achatina fulica* may have either introduced *Angiostrongylus cantonensis* into the area at the time of its arrival, or have amplified its presence if rats brought it in. If different geographic origins are clearly proven for *Achatina fulica* and *Angiostrongylus cantonensis*, *A. fulica* could not have been responsible for the introduction of the parasite. It is more likely that *Achatina fulica* played a role in the introduction of *Angiostrongylus cantonensis* if they both originated from the same country or region. Unfortunately, as no specimens of *Angiostrongylus cantonensis* have been sequenced for either New Caledonia or Barbados, it is impossible to determine whether individuals of this parasite and *Achatina fulica* in Guadeloupe originate from the same region.

## Conclusions

Our results indicate a common origin of *Achatina fulica* found on Barbados, New Caledonia and Guadeloupe, whereas *Angiostrongylus cantonensis* on Guadeloupe shares a common origin with those found in Hawaii, Brazil and Japan. However, due to a lack of information on these two species from different parts of the world, it is not possible to clearly determine the precise origin of *Angiostrongylus cantonensis* found in Guadeloupe, and if *Achatina fulica* played a role in its introduction there. In particular, sequences of *Angiostrongylus cantonensis* from Barbados and New Caledonia are needed, as are sequences of this species and *Achatina fulica* from West Africa, for which, at present, no data are available. To obtain clearer results, verify the absence of genetic variation among our samples, and enable comparison of the whole genome sequence of *Angiostrongylus cantonensis*, the present study needs to be complemented by further work in which sequences of *A. cantonensis* and *Achatina fulica* from Martinique, French Guiana, New Caledonia, and various localities in Africa are included.

## Data Availability

The 20 partial *Angiostrongylus cantonensis* cytB sequences, 27 *A. cantonensis* sequences cytC and 71 *Achatina fulica* rRNA 16S sequences have been deposited in GenBank and are available under BioSample accession numbers OQ238771–OQ238790, OQ255893–OQ255919, and OP962225–OP962295, respectively.
